# Placement into Scattered-Site or Place-Based Permanent Supportive Housing in Los Angeles County, CA, During the COVID-19 Pandemic

**DOI:** 10.1007/s10488-024-01359-1

**Published:** 2024-03-14

**Authors:** Benjamin F. Henwood, Randall Kuhn, Amanda Landrian Gonzalez, Jessie Chien, Yue Tu, Ricky Bluthenthal, Michael Cousineau, Howard Padwa, Roya Ijadi-Maghsoodi, Melissa Chinchilla, Bikki Tran Smith, Lillian Gelberg

**Affiliations:** 1 Suzanne Dworak-Peck School of Social Work, University of Southern California (USC), 669 W. 34th Street, Montgomery Ross Fisher Building, Los Angeles, CA 90089 USA; 2 Department of Community Health Sciences, Fielding School of Public Health, University of California, Los Angeles (UCLA), Los Angeles, CA USA; 3 Department of Population and Public Health Sciences, Keck School of Medicine of USC, Los Angeles, CA USA; 4 Semel Institute of Neuroscience and Human Behavior, UCLA, Los Angeles, CA USA; 5 Center for the Study of Healthcare Innovation, Implementation and Policy, VA Greater Los Angeles Healthcare System, Los Angeles, CA USA; 6 Department of Psychiatry and Biobehavioral Sciences, David Geffen School of Medicine, University of California Los Angeles, Los Angeles, CA USA; 7 VA Desert Pacific Mental Illness Research, Education, and Clinical Center, Los Angeles, CA USA; 8 Department of Biomedical & Health Sciences, University of Vermont, Burlington, VT USA; 9 Department of Family Medicine, David Geffen School of Medicine, UCLA, Los Angeles, CA USA; 10 Department of Health Policy & Management, Fielding School of Public Health, UCLA, Los Angeles, CA USA

**Keywords:** Homelessness, Housing First, Vulnerability, Gelberg-Anderson Model, Place-based Housing, Single-site Housing, Scattered-site Housing, Racial Disparities

## Abstract

There are two dominant approaches to implementing permanent supportive housing (PSH), namely place-based (PB) and scattered-site (SS). Formal guidance does not distinguish between these two models and only specifies that PSH should be reserved for those who are most vulnerable with complex health needs. To consider both system- and self-selection factors that may affect housing assignment, this study applied the Gelberg-Anderson behavioral model for vulnerable populations to compare predisposing, enabling, and need factors among people experiencing homelessness (PE) by whether they were assigned to PB-PSH (*n* = 272) or SS-PSH (*n* = 185) in Los Angeles County during the COVID-19 pandemic. This exploratory, observational study also included those who were approved but did not receive PSH (*n* = 94). Results show that there are notable differences between (a) those who received PSH versus those who did not, and (b) those in PB-PSH versus SS-PSH. Specifically, PEH who received PSH were more likely to be white, US-born, have any physical health condition, and have lower health activation scores. PEH who received PB- versus SS-PSH were more likely to be older, Black, have any alcohol use disorder, and have higher health activation scores. These findings suggest that homeless service systems may consider PB-PSH more appropriate for PEH with higher needs but also raises important questions about how race may be a factor in the type of PSH that PEH receive and whether PSH is received at all.

**Supplementary Information:**

The online version contains supplementary material available at 10.1007/s10488-024-01359-1.

## Introduction

Permanent supportive housing (PSH) is an evidence-based solution to chronic homelessness that provides immediate access to subsidized housing coupled with support services (National Academies of Sciences, Engineering, and Medicine, 2018). There are two dominant approaches to implementing PSH, namely place-based PSH (PB-PSH), referring to single-site housing placement in a specific building with other people experiencing homelessness (PEH) accompanied by on-site services, or scattered-site PSH (SS-PSH), which uses apartments rented from a private landlord to house clients while providing mobile case management services. Most guidance on housing placement for PEH does not distinguish between PB- and SS-PSH and only specifies that PSH should be reserved for those who are most vulnerable and have complex health needs (National Academies of Sciences, Engineering, and Medicine, 2018). In fact, homeless services systems have been mandated by the federal government to implement “coordinated entry systems” to connect PEH to resources and housing in the most efficient and equitable way possible but do not specify whether PB- and SS-PSH should be considered a different type of resource or intervention model.

Research related to the comparative effectiveness of PB-PSH versus SS-PSH is limited and has been mixed (Dickson-Gomez et al., 2021). Qualitative research has suggested PB-PSH may provide more supportive services (Henwood et al., 2018) and be more effective than SS-PSH in improving disability severity, community integration, and recovery (Somers et al., 2017). Yet housing preferences may complicate comparative effectiveness research on PSH (Dickson-Gomez et al., 2020), especially since recent studies have suggested that PEH have strong preferences for specific housing characteristics (e.g., location, private bedroom or bathroom, allows pets, etc.) that may influence whether PEH accept placement into either PB- or SS-PSH (Kuhn et al., 2022; Ward et al., 2022). Indeed, a meta-analysis of eight studies with more than 3,000 participants found that 84% of PEH with mental disorders preferred SS-PSH (Richter & Hoffmann, 2017), despite greater feelings of social isolation than those in PB-PSH.

In addition to how PEH preferences may influence housing placement, concerns also persist about how scarce PSH resources are allocated to clients and the potential of selection bias based on demographic characteristics such as age, sex, gender, and race and ethnicity (Brown et al., 2018; Cronley, 2022). While recent work supported by the Los Angeles Homeless Services Authority found no evidence of racial disparities in PSH placements, the study did not consider differences in the type of PSH placement (Edwards et al., 2021). This may be an important consideration given that the same study found that black PEH had fewer housing options because of racial discrimination by landlords and even homeless service system staff, and that ultimately Black PSH residents were 19% more likely to return to homelessness than white residents (Edwards et al., 2021).

In the current exploratory study, we seek to understand whether there are differences between those placed in PB- versus SS-PSH and consider what may be driving those differences, using cross-sectional data from a convenience sample of PEH who were approved for PSH through the Los Angeles County coordinated entry system. This was undertaken as part of a larger study known as the Person-Centered Housing Options, Outcomes, Services, & Environment (PCHOOSE) study, which was designed to compare the effectiveness of PB- versus SS-PSH during the COVID-19 pandemic regarding patient-centered and COVID-19-related behaviors and outcomes using a longitudinal observational design (Henwood et al., 2023). Recruitment for the study occurred during 18 months between January 2021 and July 2022 in Los Angeles County, California, which witnessed a particularly intensive rehousing effort due to the COVID-19 pandemic and the construction of many new housing units through a voter-supported bond measure aimed at reducing the size and addressing the needs of one of the nation’s largest unhoused populations. PEH qualified for the study based on having been approved for PSH through the Los Angeles County coordinated entry system and had either moved into PSH in the preceding 2 weeks or were expected to move into housing within 30 days. Since not everyone who was approved for PSH had moved in at the start of the study, we ended up with a group of participants who could not be classified as having received either PB- or SS-PSH because they never moved in during our study period.

**Fig. 1:**
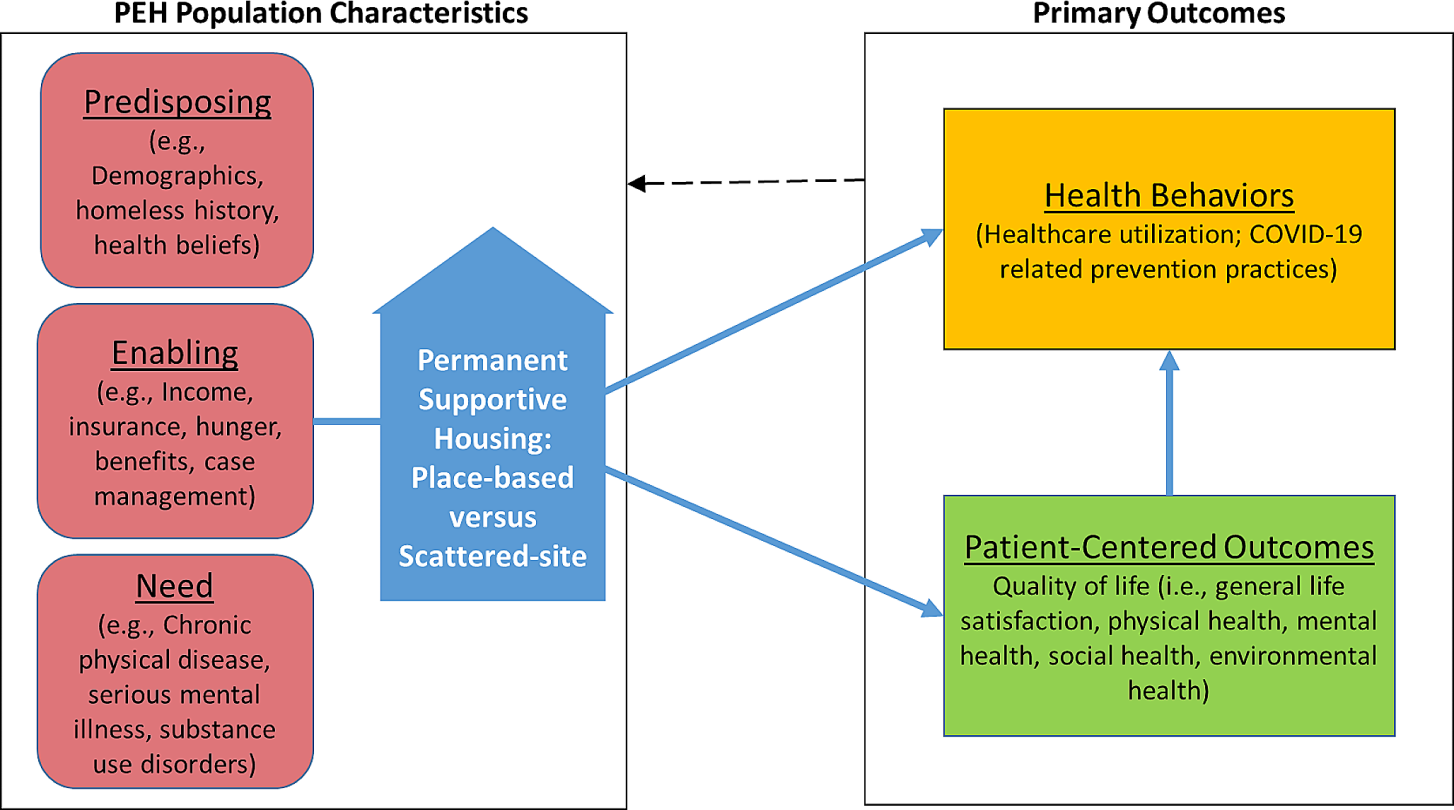
Gelberg-Anderson behavioral model for vulnerable populations (modified for permanent supportive housing and COVID-19)

To consider both system- and self-selection factors that may affect placement into PSH among other factors, the PCHOOSE study applied the Gelberg-Anderson Behavioral Model for Vulnerable Populations (Gelberg et al., 2000), as depicted in Fig. 1, which considers predisposing, enabling, and need factors to understand variations in health behaviors. For this study, we consider housing preferences, which is not typically included in the model, to be a predisposing factor. Using survey data from participants when they initially enrolled in the PCHOOSE study, we examined the following study questions:

1. Given that everyone who enrolled in the study had been approved for PSH through the Los Angeles County coordinated entry system, are there differences between those who obtained PSH compared to those who did not end up obtaining PSH in terms of predisposing, enabling, and need factors?
2. Are there differences between those placed in PB- versus SS-PSH in terms of predisposing, enabling, and need factors?
3. Do any of these patterns vary by respondent sex, gender, or race and ethnicity?

## Methods

### Recruitment and data Collection

Recruitment into the PCHOOSE study began in January 2021 amid a significant surge of COVID-19 infections in Los Angeles during the first year of the pandemic, which precluded in-person enrollment, and ended in July 2022. Recruitment depended on housing case managers already interacting with PEH as part of the housing placement process who informed anyone approved for PSH about the study. Given feedback that rapid rehousing programs that typically provide short-term rental assistance were used during the pandemic to serve people who qualified for PSH by pairing these placements with more intensive supportive services, we included rapid rehousing as part of SS-PSH if it included services. Both PB- and SS-PSH were otherwise clearly defined in the homeless services system.

PEH were eligible to participate in the study if they were 18 years old or older, had been approved for PSH through the Los Angeles County coordinated entry system, had either moved into housing in the preceding 2 weeks or expected to move into housing in 30 days, could be interviewed in English or Spanish, and could provide informed consent. Approval for PSH in Los Angeles County typically includes documentation of having experienced chronic homelessness and having disabilities, chronic medical conditions, and/or behavioral health conditions. Case managers across 26 housing agencies that placed PEH in PSH helped those interested in the study in setting up a meeting with the study staff via phone or Zoom to complete the enrollment process, which included obtaining informed consent using an electronic signature. Although this process remained largely intact through the recruitment period that ended in July 2022, in-person recruitment by study staff occasionally occurred when COVID-19 rates were low and visitors were allowed at housing program sites.

Upon enrollment in the study, participants received smartphones with study-paid unlimited talk, text, and data plans that were used to send links to a web-based survey via text message. Participants first self-administered and completed a 20-minute survey to capture basic demographic and historical information about housing and health. To reduce the burden involved in completing a lengthy questionnaire at the time of enrollment, a follow-up survey link was sent approximately 1 day later to collect additional baseline outcome measures. In total, 563 participants were enrolled and completed a baseline survey. Given the uncertainties of the housing process even after being approved for PSH, as mentioned earlier our study included PEH who were approved for PSH but never assigned to a PSH unit during the study period that ended in June 2023.

Surveys available in English or Spanish were administered using a web-based survey distributed via text message. Respondents who did not respond or requested support could answer the survey by phone interview, with answers entered by the survey staff. To reduce respondent fatigue and reduce the risk of re-traumatization, and in response to feedback from stakeholders with lived experience, the survey employed a trauma-informed design featuring plain language, readable font size, and extensive buffering language to prepare respondents for difficult questions. Furthermore, while all survey questions required a response from participants for them to proceed, all questions included a “prefer not to answer” option, which was deemed critical to reducing respondent burden but resulted in a relatively high rate of missingness. Participants received a $15 electronic gift card incentive for any completed survey. All study protocols were approved by the [blinded] Institutional Review Board; more details about the study design and procedures can also be found in [blinded].

### Measures

The baseline survey covered various topics and included information on demographics, systems involvement, housing needs, and health characteristics. We describe the measures included in this analysis in relation to our conceptual framework (Fig. 1), grouping various characteristics by predisposing, need, and enabling factors. A complete list of survey measures and response options as presented to participants are provided as a Supplementary Information file.

### Dependent Variable

Our dependent variable for this study was type of housing placement, which included placement in PB-PSH or SS-PSH or not assigned to a PSH unit (i.e., unassigned PSH). We noted that 82% of participants who were assigned a PB- or SS-PSH unit had already moved in upon study enrollment, whereas the remaining 18% of participants moved in an average of 2 months later. The group not assigned to a housing unit had been approved for PSH at least one year prior to their study enrollment date.

### Independent Variables

Predisposing demographic factors included age, gender identity, sexual orientation, relationship status, veteran status, and criminal justice involvement. Age was a continuous variable calculated from participants’ birth year. Participants were asked what gender they identify as, with answers used to create a binary variable categorizing gender identity as man versus woman or other. Sexual orientation was dichotomized as heterosexual or straight versus lesbian, gay, bisexual, or other sexual orientation (LGB+). Current relationship status was categorized as single; currently married or in a domestic partnership; and separated, divorced, or widowed, whereas veteran status was captured by asking participants if they ever served in the military (yes or no). Criminal justice involvement (i.e., ever spending time in jail, prison, or a juvenile detention center) was a categorical variable coded as (1) no history of involvement, (2) a history but not currently on parole or probation, and (3) a history and currently on parole or probation.

Predisposing social structure factors consisted of race and ethnicity, country of birth, educational attainment, employment status, family structure characteristics, and homelessness history. Participants were asked to self-identify their race and ethnicity as: (a) Black or African American, (b) White, (c) Hispanic or Latino, (d) Asian American, (e) Native Hawaiian or Pacific Islander, (f) Native American or Alaska Native, (g) multiracial or multiethnic, or (h) other. Due to small sample sizes, responses were categorized into non-Hispanic Black or African American (hereafter referred to as “Black”), non-Hispanic White (hereafter referred to as “White”), Latino or Hispanic, and other or multiracial. Country of birth was a dichotomous variable indicating whether participants were foreign- or U.S.-born. Educational attainment was measured as the highest education level completed and categorized as less than high school, high school graduate or GED, some college, or associate or bachelor’s degree or higher. Categories of current employment status included employed (either full-time or part-time), unemployed, or retired. Family structure characteristics encompassed whether participants had any children younger than 18 living with them (yes or no). Participants also responded to several questions related to their homelessness history, including the total time they had experienced homelessness in their life (categorized as less than 5 years, 5–9 years, 10–19 years, and 20 years or more). They were then prompted to select all the places they have ever stayed. Drawing from the U.S. Housing and Urban Development’s definition of homelessness, a binary variable was created to capture whether a participant had a history of unsheltered homelessness, defined as ever staying in an abandoned building; bus, subway, or train; car, truck, van, or RV; garage or shed not meant for living; indoor public place; or outside on the street, park or beach. Last, a variable was created to capture if participants were unsheltered in the month leading up to the start of the COVID-19 pandemic in March 2020.

Predisposing health beliefs were operationalized as health activation and housing preferences. Health activation was measured using four items from the Insignia Health Patient Activation Measure. Each item is rated on a 4-point Likert scale ranging from 0 (*strongly disagree*) to 3 (*strongly agree*) and evaluates a person’s knowledge, skills, and confidence to manage their health. Responses were averaged across the four items to generate a composite score ranging from 0 to 3, with higher values indicating greater patient activation (Cronbach’s α = 0.74). Four items regarding housing preferences were also assessed and used to create variables capturing participants’ preferences for living (a) alone or with a partner, spouse, or family member versus with roommates; (b) in a setting where most residents have experienced homelessness versus most residents having no homelessness experiences; (c) in a sober living setting versus not requiring sober living; and (d) with people of the same gender versus no gender requirement. Participants also had the option to indicate no preference in these four categories.

Enabling factors included questions related to receiving government benefits, monthly income, and health insurance status. Participants were asked whether they received any of the following government benefits: (a) General Relief, Temporary Cash Assistance, Family Independence Temporary Assistance, or Family Investment Program; (b) Social Security Disability Income or Social Security Income; (c) retirement income; (d) Supplemental Nutritional Assistance, CalFresh, or WIC; (e) Temporary Assistance for Needy Families; (f) veterans’ benefits; (g) cash aid and services to eligible families that have a child or children in the home (CalWORKS); (h) unemployment benefits; (i) federal COVID-19 paycheck protection or cash relief; (j) other; or (k) none of the above. For the regression analysis, we generated indicator variables of whether a participant received each benefit. Participants were also asked to provide an estimate of their total monthly income, categorized as less than $500, $500–$900, $1,000–$1,999, and $2,000 or more. Health insurance status was a binary variable of whether participants reported having health insurance that helps pay for some or all of their health care costs.

Need factors included health status (i.e., physical, mental, and substance use), trauma history, and perceived housing needs. Binary variables capturing any physical and mental health condition were created by asking participants to select physical health conditions they had ever been diagnosed with from the list provided in the Centers for Disease Control and Prevention’s COVID-19 risk factor screener (e.g., serious heart condition, diabetes, asthma, cancer), followed by diagnosed mental health conditions (e.g., anxiety, bipolar disorder, major depression, PTSD, schizophrenia, other). Participants also indicated if they had ever been diagnosed with a substance use disorder, capturing whether they endorsed alcohol or drug use (i.e., cannabis, opioid, stimulant), separately. Trauma history was a categorical variable generated using responses to PC-PTSD-5, a screening measure for probable PTSD (Bovin et al., 2021; Prins et al., 2016). Participants were first asked whether they had ever experienced a traumatic event. Those who answered “yes” were then prompted to answer five questions about past-month experiences of PTSD-related symptoms (e.g., being watchful, feeling detached). Participants who indicated experiencing three or more PTSD symptoms in the past month were defined as having PTSD. A consolidated measure was then constructed that distinguished among respondents who (a) had not experienced a traumatic event, (b) had experienced a traumatic event but did not have PTSD in the past month, and (c) had experienced a traumatic event and had PTSD in the past month. Finally, housing needs were assessed by asking participants to select their requirements from the following list: (a) allowed to stay with partner, spouse, or family member; (b) handicap accessible; (c) in a particular neighborhood; (d) allowed to stay with pets; (e) a lot of space to store possessions; (f) live with other veterans; (g) other; and (h) none of the above. Indicator variables were generated for each housing need option.

### Data Analysis

We first removed participants with missingness in key predisposing characteristics (i.e., age, sex, gender, and race and ethnicity) or had more than eight “prefer not to answer” responses. We note that the latter was treated as missing for categorical variables, except for sex, gender, race and ethnicity, and trauma and PTSD history. We then assessed the representativeness of the PCHOOSE sample relative to all PSH placements recorded in Los Angeles County administrative records from April 2021 through September 2022 (*N* = 7,231), a period that roughly overlapped the study recruitment period. Our samples and the County population of all PSH placements in the relative share of PB- vs. SS-PSH placements and demographic composition across gender, race and ethnicity, and age were largely similar other than our sample had a higher percentage of female (49% vs. 40%) and non-Hispanic White (34% vs. 18%) in SS-PSH as compared to the overall County (results included as a Supplementary Information file). We also considered differences in our SS-PSH for those who received rapid rehousing with support services (*n* = 114 or 62% of those in SS-PSH); these individuals were more likely than other SS-PSH to be unemployed (83% vs. 64%), more likely to have co-residing children (19% vs. 7%), less likely to have a history of unsheltered homelessness (87% vs. 97%), and less likely to have health insurance (87% vs. 99%), but otherwise did not differ from others in the SS-PSH sample as indicated in the Supplementary Information file.

Descriptive statistics of selected measurements from our samples are presented as frequencies and column percentages for categorical variables and means and standard deviations for continuous variables. To test the unadjusted associations between the measured characteristics and PSH placement (PB-PSH vs. SS-PSH vs. unassigned to PSH), Pearson’s chi-square test or Fisher’s exact test were used for categorical variables if at least one expected cell count in the cross table was less than 5, whereas the Kruskal-Wallis rank sum test was used for continuous variables.

Eight variables to be used in regression models had missing responses; to replace missing responses based on the missing at random assumption, multiple imputation methods were used: predictive mean matching for continuous variables, logistic regression for dichotomous variables, multinomial logistic regression for unordered categorical variables, and ordinal logistic regression for ordered categorical variables. Five imputed complete datasets were generated. Using the same bivariate statistical tests previously described, a robustness check was conducted to assess whether any characteristics statistically significantly differed between the non-imputed and imputed datasets. No differences were detected based on significance level of 0.05; therefore, the imputed datasets were used to complete the regression analyses.

Two multivariable logistic regression models were fitted for PSH assigned versus unassigned arm and PB-PSH versus SS-PSH using the pooled imputed datasets. Key variables of interest and those with *p*-values less than or equal to 0.05 in bivariate analyses were included in both logistic regression models. Multicollinearity was examined by the variance inflation factor, whereby variables with larger variance inflation factors were removed from the model. This resulted in the removal of any substance use disorder, which was found to be highly correlated with any drug use disorder. Statistical significance was assessed at a significance level of *p* < .05; however, to reduce the risk of potential type I error inflation due to multiple testing, statistical significance was also assessed using a Bonferroni correction of *p* < .002, calculated by dividing 0.05 by the number of hypotheses tested in the model(s) (i.e., 29). All statistical analyses were performed in R version 4.3.0.

## Results

**Fig. 2:**
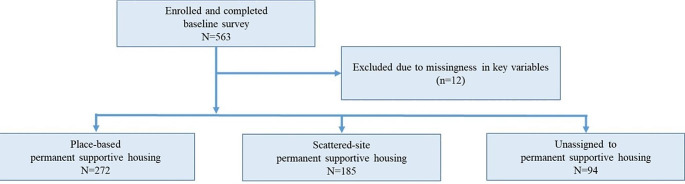
Flowchart of participants (CONSORT diagram)

As depicted in Figs. 2 and 563 individuals enrolled in the study and completed a baseline demographic survey. Thirteen of those participants were excluded from analyses due to missingness in key variables, resulting in a total analytic sample of 550 PEH. Our sample included 272 PEH in PB-PSH, 185 in SS-PSH, and 94 not been assigned to a PSH unit.

### Descriptive Characteristics and Bivariate Findings

The characteristics of the total sample and by PB-PSH, SS-PSH, or unassigned at the time of enrollment are shown in Table 1 and organized by predisposing (i.e., health beliefs, demographics, and social structure), enabling (i.e., monthly income, health insurance, government benefits), and need (i.e., physical health, mental health, substance use disorder, trauma history, housing needs) factors.

**Table 1:** Predisposing, enabling, and need factors in the total sample and by PSH groups

Characteristic	Total	PSH assignment^a^			
		PB-PSH	SS-PSH	Unassigned	
	(*N* = 551)	(*n* = 272)	(*n* = 185)	(*n* = 94)	
	*n* (%)^b^	*n* (%)^b^	*n* (%)^b^	*n* (%)^b^	*p* ^c^
*Predisposing factors*					
Demographic characteristics					
Age (years), *M* (*SD*)	47.97 (14.38)	48.99 (14.29)	45.55 (14.64)	49.78 (13.63)	0.02
Gender identity					0.03
Man	321 (58.26)	165 (60.66)	94 (50.81)	62 (65.96)	
Woman or other	230 (41.74)	107 (39.34)	91 (49.19)	32 (34.04)	
Sexual orientation					0.03
Heterosexual or straight	471 (87.71)	227 (85.02)	155 (87.08)	89 (96.74)	
LGB+	53 (9.87)	33 (12.36)	18 (10.11)	2 (2.17)	
Prefer not to answer	13 (2.42)	7 (2.62)	5 (2.81)	1 (1.09)	
Relationship status					< 0.01
Single	371 (68.83)	188 (71.76)	112 (61.20)	71 (75.53)	
Married or domestic partnership	53 (9.83)	14 (5.34)	31 (16.94)	8 (8.51)	
Separated, divorced, or widowed	115 (21.34)	60 (22.90)	40 (21.86)	15 (15.96)	
Veteran of the military (yes)	35 (6.36)	29 (10.70)	5 (2.70)	1 (1.06)	< 0.01
Criminal justice involvement					0.50
None	217 (40.04)	99 (37.08)	82 (45.05)	36 (38.71)	
Prior involvement	282 (52.03)	147 (55.06)	85 (46.70)	50 (53.76)	
On parole or probation	43 (7.93)	21 (7.87)	15 (8.24)	7 (7.53)	
Social structure					
Race and ethnicity					0.01
Black	206 (37.39)	114 (41.91)	56 (30.27)	36 (38.30)	
White	133 (24.14)	54 (19.85)	61 (33.51)	17 (18.09)	
Hispanic or Latino	138 (25.05)	66 (24.26)	47 (25.41)	25 (26.60)	
Other or multiracial	74 (13.43)	38 (13.97)	20 (10.81)	16 (17.02)	
Foreign-born (yes)	54 (9.80)	19 (6.99)	19 (10.27)	16 (17.02)	0.02
Educational attainment					0.53
Less than high school	141 (25.82)	78 (29.00)	39 (21.20)	24 (25.81)	
High school or GED	172 (31.50)	85 (31.60)	60 (32.61)	27 (29.03)	
Some college	158 (28.94)	71 (26.39)	56 (30.43)	31 (33.33)	
Associate or bachelor’s degree or higher	75 (13.74)	35 (13.01)	29 (15.76)	11 (11.83)	
Employment status					0.16
Employed full- or part-time	57 (10.86)	21 (8.14)	24 (13.33)	12 (13.79)	
Unemployed	392 (74.67)	197 (76.36)	136 (75.56)	59 (67.82)	
Retired	76 (14.48)	40 (15.50)	20 (11.11)	16 (18.39)	
Co-residing children < 18 years old (yes)	50 (9.14)	22 (8.15)	26 (14.13)	2 (2.15)	< 0.01
Years homeless					< 0.01
Less than 5	219 (39.89)	93 (34.32)	90 (48.65)	36 (38.71)	
5–9	138 (25.14)	70 (25.83)	52 (28.11)	16 (17.20)	
10–19	114 (20.77)	67 (24.72)	28 (15.14)	19 (20.43)	
20 or more	78 (14.21)	41 (15.13)	15 (8.11)	22 (23.66)	
History of unsheltered homelessness	486 (90.00)	234 (87.97)	165 (91.16)	87 (93.55)	0.25
Unsheltered homelessness during month before COVID-19 pandemic onset	314 (57.61)	154 (57.46)	104 (56.83)	56 (59.57)	0.91
Health beliefs					
Health activation score,^d^*M* (SD)	2.04 (0.49)	2.07 (0.50)	1.93 (0.41)	2.16 (0.56)	< 0.01
Living alone or with roommates					0.02
Alone or with spouse or family member	480 (92.66)	237 (94.05)	159 (92.44)	84 (89.36)	
With roommate	29 (5.60)	13 (5.16)	12 (6.98)	4 (4.26)	
No preference	9 (1.74)	2 (0.79)	1 (0.58)	6 (6.38)	
Living with homeless residents					< 0.01
Most residents with homeless experience	89 (16.70)	57 (22.01)	24 (13.19)	8 (8.70)	
Most residents with no homeless experience	136 (25.52)	53 (20.46)	49 (26.92)	34 (36.96)	
No preference	308 (57.79)	149 (57.53)	109 (59.89)	50 (54.35)	
Require sober living housing (yes)	59 (19.60)	15 (17.65)	23 (17.69)	21 (24.42)	0.41
Requires same gender housing (yes)	21 (6.98)	5 (5.88)	7 (5.38)	9 (10.47)	0.41
*Enabling factors*					
Receive any type of benefits (yes)	486 (88.20)	246 (90.44)	160 (86.49)	80 (85.11)	0.26
Monthly income					0.11
Less than $500	282 (51.18)	145 (53.31)	80 (43.24)	57 (60.64)	
$500–$999	126 (22.87)	63 (23.16)	48 (25.95)	15 (15.96)	
$1,000–$1,999	117 (21.23)	54 (19.85)	46 (24.86)	17 (18.09)	
$2,000 or more	26 (4.72)	10 (3.68)	11 (5.95)	5 (5.32)	
Have health insurance (yes)	502 (92.11)	253 (91.30)	168 (91.30)	81 (87.10)	0.07
*Need factors*					
Any physical health condition (yes)	367 (68.60)	190 (72.80)	123 (67.58)	54 (58.06)	0.03
Any mental health condition (yes)	367 (67.96)	206 (77.44)	107 (59.12)	54 (57.45)	< 0.01
Any drug use disorder (yes)	98 (17.79)	53 (19.49)	29 (15.68)	16 (17.02)	0.57
Alcohol use disorder (yes)	72 (13.07)	53 (19.12)	12 (6.49)	8 (8.51)	< 0.01
Trauma history and PTSD symptoms					0.23
None	143 (25.95)	67 (24.63)	52 (28.11)	24 (25.53)	
Trauma but no PTSD symptoms	159 (28.86)	80 (29.41)	54 (29.19)	25 (26.60)	
Trauma and PTSD symptoms	194 (35.21)	97 (35.66)	56 (30.27)	41 (43.62)	
Prefer not to answer	55 (10.00)	28 (10.29)	23 (12.43)	4 (4.26)	
Housing needs (yes)					
Spouse, partner, or family member allowed	215 (39.02)	94 (34.56)	80 (43.24)	41 (43.62)	0.11
Pets allowed	208 (37.75)	102 (37.50)	59 (31.89)	47 (50.00)	0.01
Lot of space for possessions	184 (33.39)	98 (36.03)	54 (29.19)	32 (34.04)	0.31
Private bathroom	176 (58.47)	42 (49.41)	76 (58.46)	58 (67.44)	0.06
Handicap accessible	145 (26.32)	76 (27.94)	41 (22.16)	28 (29.79)	0.27
Particular neighborhood	143 (25.95)	54 (19.85)	57 (30.81)	32 (34.04)	< 0.01
Other	58 (10.53)	29 (10.66)	23 (12.43)	6 (6.38)	. 30
None of the above	92 (16.70)	52 (19.12)	31 (16.76)	9 (9.57)	0.10
Prefer not to answer	26 (4.72)	19 (6.99)	6 (3.24)	1 (1.06)	0.04

Note. LGB + = lesbian, gay, bisexual, or other sexual orientation; PB-PSH = place-based permanent supportive housing; PSH = permanent supportive housing; SS-PSH = scattered-site permanent supportive housing. Percentages may not add to 100 due to rounding

^a^ As specified at the time of enrollment

^b^ Unless otherwise noted

^c^ Kruskal-Wallis rank sum test, Fisher’s exact test for count data with simulated *p*-value (based on 10,000 replicates), or Pearson’s chi-square test

^d^ Possible health activation scores range from 0–3, with higher values indicating greater patient activation

### Predisposing Factors

Regarding demographic characteristics, PEH in SS-PSH were slightly younger than those in PB-PSH or unassigned, with an average age of about 46 years in SS-PSH compared to 49 and 50 years in PB-PSH and unassigned (*p* = .02). Significant differences were also observed across groups in gender identity and sexual orientation; more than 60% of PEH assigned to PB-PSH (61%) and those unassigned PSH (66%) identified as men compared to 50% of PEH assigned to SS-PSH (*p =* .03). A significantly lower proportion of those unassigned to PSH identified as LGB + than those in the other two study groups (2.2% vs. 12.4% and 10.1% among those in PB-PSH and SS-PSH groups, respectively; *p =* .03). A significantly higher proportion of unassigned PEH were single (75.5% vs. 71.8% and 61.2% among PB-PSH and SS-PSH, respectively; *p* < .01). PEH in PB-PSH were most likely to have served in the military compared to PEH in SS-PSH or unassigned (10.7% vs. 2.7% and 1.1%, respectively; *p* < .01). The majority of PEH in the total sample had prior criminal justice involvement (52.1%), with no statistically significant differences across the three study groups.

Regarding social structure characteristics, the highest proportion of PEH in the sample completed high school (31.5%), were unemployed (74.7%), had a history of unsheltered homelessness (90.0%), and were unsheltered in the month prior to the onset of the COVID-19 pandemic (57.6%), with no statistically significant differences across the three study groups. PEH in PB-PSH were more likely to be Black than those in SS-PSH and unassigned (41.9% vs. 30.3% and 38.3%, respectively; *p* = .01), whereas those in SS-PSH were more likely to be White (33.5% vs. 19.9% in PB-PSH and 18.1% of unassigned PEH; *p* = .01). 17% of PEH unassigned to PSH were foreign-born compared to 7% and 10% of PEH assigned to PB-PSH and SS-PSH, respectively (*p =* .02). PEH in SS-PSH were the most likely to have children younger than 18 years living with them (14.1% vs. 8.2% in PB-PSH and 2.2% of unassigned PEH; *p* < .01) and to have been homeless for less than 5 years (48.7% vs. 34.3% and 38.7%, respectively; *p* < .01).

Regarding health beliefs, PEH assigned to SS-PSH had lower average health activation scores than those assigned to PB-PSH or unassigned, indicating less knowledge, skills, and confidence in managing their health (1.9 vs. 2.1 and 2.2, respectively; *p* < .01). Differences in housing preferences across the three groups were also present. PEH unassigned to PSH were the most likely to indicate no preference for living alone or with roommates (6.4% vs. 1.7% and 0.6% in PB-PSH and SS-PSH, respectively; *p =* .02). PEH unassigned to PSH were also the most likely to prefer housing where most residents had no homelessness experience (37.0% vs. 20.5% in PB-PSH and 26.9% in SS-PSH; *p* < .01). Finally, unassigned PEH were most likely to prefer sober living facilities (24.4% vs. 17.7% in both PB-PSH and SS-PSH) and living with others of the same gender (10.5% vs. 5.9% in PB-PSH and 5.4% in SS-PSH), although these differences were not statistically significant.

### Enabling Factors

Most PEH in this sample reported receiving any type of public benefits (88.2%) and having health insurance (92.1%), and more than half (51.2%) reported a monthly income of less than $500. No statistically significant differences in these characteristics occurred across study group.

### Need Factors

More PEH in PB-PSH, compared to those in SS-PSH or unassigned PSH, reported any physical health condition (72.8% vs. 67.6% and 58.1%, respectively; *p* = .03), any mental health condition (77.4% vs. 59.1% and 57.5%; *p* < .01), or any alcohol use disorder (19.1% vs. 6.5% and 8.5%; *p* < .01). 35% of PEH in the total sample reported having ever experience trauma and having current PTSD symptoms, with no statistically significant differences across study groups. Finally, the most common housing needs in this sample were allowances for a spouse, partner, or family member (39.0%) or pet (37.8%) to live with them, and having adequate space for possessions (33.4%). A significantly higher proportion of PEH unassigned to PSH indicated a requirement for pet allowances than those in the other two housing groups (50.0% vs. 37.5% in PB-PSH and 31.9% in SS-PSH; *p =* .01). Lastly, PEH in SS-PSH and unassigned PEH were significantly more likely than PEH in PB-PSH to identify a need for living in a particular neighborhood (30.8% in SS-PSH and 34.0% in unassigned vs. 19.9% in PB-PSH; *p* < .01).

### Multivariable Logistic Regression Analyses

The results of multivariable logistic regression models examining the association between predisposing, enabling, and need factors and (a) assigned versus unassigned to PSH and (b) PB-PSH versus SS-PSH are provided in Table 2.

**Table 2:** Multivariable logistic regression examining the association between predisposing, enabling, and need factors and PSH assignment

Characteristic	Assigned vs. unassigned^a^			PB-PSH vs. SS-PSH^b^			
	aOR	95% CI	*p*	aOR	95% CI	*p*	
*Predisposing factors*							
Age (years)	0.99	0.97, 1.01	0.27	1.03	1.01, 1.05	< 0.01	
Gender identity (ref = man)							
Woman or other	1.13	0.65, 1.98	0.66	0.76	0.47, 1.23	0.26	
Race and ethnicity (ref = Black)							
White	2.22	1.05, 4.70	0.04	0.32	0.18, 0.59	< .01^c^	
Hispanic or Latino	1.23	0.62, 2.46	0.55	0.88	0.49, 1.58	0.66	
Other or multiracial	0.93	0.43, 2.03	0.86	0.96	0.46, 2.02	0.92	
Foreign-born (ref = U.S.-born)	0.29	0.13, 0.66	< 0.01	0.86	0.37, 1.99	0.73	
Relationship status (ref = single)							
Married or domestic partnership	1.58	0.62, 4.00	0.34	0.39	0.18, 0.85	0.02	
Separated, divorced, or widowed	1.78	0.88, 3.61	0.11	0.70	0.40, 1.25	0.23	
Co-residing children < 18 years old (ref = no)	8.78	1.77, 43.42	< 0.01	1.30	0.58, 2.93	0.52	
Years homeless (ref = less than 5)							
5–9	1.22	0.60, 2.46	0.59	1.25	0.27, 2.16	0.43	
10–19	0.72	0.35, 1.48	0.38	1.57	0.82, 3.01	0.17	
20 or more	0.39	0.18, 0.84	0.02	1.72	0.78, 3.80	0.18	
History of unsheltered homelessness (ref = no)	0.80	0.27, 2.39	0.69	0.93	0.41, 2.09	0.86	
Health activation score	0.33	0.19, 0.60	< .01^c^	2.22	1.34, 3.70	< 0.01	
Housing and shelter preference (ref = none)							
Most residents with homeless experience	1.33	0.55, 3.25	0.53	1.57	0.84, 2.96	0.15	
Most residents with no homeless experience	0.62	0.36, 1.09	0.10	0.89	0.52, 1.51	0.66	
*Enabling factors*							
SNAP, CalFresh, or WIC benefits (ref = no)	0.54	0.32, 0.90	0.02	0.75	0.48, 1.18	0.22	
*Need factors*							
Any physical health condition (ref = no)	2.18	1.23, 3.86	< 0.01	1.03	0.61, 1.74	0.90	
Any mental health condition (ref = no)	1.78	1.01, 3.13	0.05	1.87	1.11, 3.15	0.02	
Any alcohol use disorder (ref = no)	2.20	0.90, 5.36	0.08	3.21	1.48, 6.97	< 0.01	
Any drug use disorder (ref = no)	0.80	0.40, 1.62	0.54	1.29	0.69, 2.41	0.43	
Prefer not to answer substance use disorder question (ref = no)	2.29	0.26, 19.86	0.54	1.79	0.62, 5.19	0.28	
Housing needs							
Spouse, partner, or family member allowed (ref = no)	0.74	0.41, 1.33	0.32	0.83	0.49, 1.41	0.49	
Handicap accessible (ref = no)	0.97	0.53, 1.79	0.93	1.36	0.77, 2.40	0.28	
Particular neighborhood (ref = no)	0.65	0.36, 1.16	0.14	0.53	0.31, 0.92	0.02	
Pets allowed (ref = no)	0.43	0.24, 0.79	< 0.01	1.83	1.07, 3.12	0.03	
Space for possessions (ref = no)	1.42	0.78, 2.56	0.25	1.18	0.69, 2.00	0.55	
Live with other veterans (ref = no)	2.28	0.42, 12.31	0.34	0.86	0.21, 3.51	0.83	
Prefer not to answer (ref = no)	3.88	0.42, 35.70	0.23	2.15	0.69, 6.73	0.19	

Note. PB-PSH = place-based permanent supportive housing; PSH = permanent supportive housing; SS-PSH = scattered-site permanent supportive housing; aOR = adjusted odds ratio; 95% CI = 95% confidence interval; ref = reference category

^a^ Total *N* = 551 (assigned: *n* = 457; unassigned: *n* = 94)

^b^ Total *N* = 457 (PB-PSH: *n* = 272; SS-PSH: *n* = 185)

^c^ Association remains statistically significant under Bonferroni correction of *p* < .002

### Predictors of PSH Assignment Status (Assigned versus Unassigned)

Holding all other factors constant, having co-residing children younger than 18 (aOR = 8.78, 95% CI [1.77, 43.42]) and having any physical health condition (aOR = 2.18, 95% CI [1.23, 3.86]) were each associated with statistically significant increased odds of being assigned to a PSH unit. Further, PEH who identified as White also had a significantly higher odds of being assigned to a PSH unit than those who identified as Black (aOR = 2.22, 95% CI [1.05, 4.70]). Alternatively, a higher health activation score (i.e., greater knowledge, skills, and confidence in managing personal health) was associated with statistically significantly lower odds of being assigned to a PSH unit versus unassigned (aOR = 0.33, 95% CI [0.19, 0.60]). This association remained statistically significant under a Bonferroni correction of *p* < .002. Foreign-born PEH had roughly 70% lower odds of being assigned to a PSH unit than U.S.-born PEH (aOR = 0.29, 95% CI [0.13, 0.66]). Compared to PEH who were homeless for less than 5 years, those who had been homeless for 20 or more years had about 60% lower odds of being assigned to a PSH unit (aOR = 0.39, 95% CI [0.18, 0.84]). Finally, PEH who reported receiving SNAP, CalFresh, or WIC benefits (aOR = 0.54, 95% CI [0.32, 0.90]) and needing pet allowances (aOR = 0.43, 95% CI [0.24, 0.79]) also had statistically significantly lower odds of being assigned a PSH unit.

### Predictors of Being Assigned to PB-SPH versus SS-PSH

A higher health activation score (aOR = 2.22, 95% CI [1.34, 3.70]) and higher age (aOR = 1.03, 95% CI [1.01, 1.05]) were each associated with increased odds of being assigned to PB-PSH versus SS-PSH. Further, PEH with any mental health condition had nearly twice the odds of being assigned to PB-PSH versus SS-PSH than those who had no mental health condition (aOR = 1.87, 95% CI [1.11, 3.15]), whereas those reporting any alcohol use disorder had more than three times the odds of being assigned to PB-PSH versus SS-PSH (aOR = 3.21, 95% CI [1.48, 6.97]). Compared to Black PEH, White PEH had statistically significantly lower odds of being assigned to PB-PSH versus SS-PSH (aOR = 0.32, 95% CI [0.18, 0.59]). This association remained statistically significant under a Bonferroni correction of *p* < .002. PEH who were married or in a domestic partnership had about 60% lower odds than single PEH of being assigned to PB-PSH versus SS-PSH (aOR = 0.39, 95% CI [0.18, 0.85]). Last, PEH needing pet accommodations had significantly higher odds of being assigned to PB-PSH versus SS-PSH (aOR = 1.84, 95% CI [1.06, 3.19]), whereas those with preferences for a particular neighborhood had significantly lower odds of being assigned to PB-PSH versus SS-PSH (aOR = 0.31; 95% CI [0.31, 0.92].

## Discussion

The results of this exploratory study, which used cross-sectional data from a convenience sample of PEH who were approved for PSH through the Los Angeles County coordinated entry system during the COVID-19 pandemic, suggest that there are notable differences between (a) those who eventually end up in a housing unit versus those who do not, and (b) between those who end up in PB-PSH versus SS-PSH. Perhaps most strikingly, among PEH approved for PSH, Black PEH were less likely than White PEH to end up in a housing unit, and for those who did receive housing, Black PEH were more likely than White PEH to live in PB-PSH rather than SS-PSH. The former runs contrary to a previous system-wide analysis that was conducted prior to the pandemic and did not find racial disparities in PSH placement (Edwards et al., 2021), which could be differently interpreted. First, it raises the possibility that the housing placement process was differently implemented during the pandemic that introduced differences based on race. Second, it could be that distinguishing between those approved for PSH versus those who received PSH is a meaningful distinction that should be more carefully considered and may help explain the previous finding that Black PSH residents in Los Angeles were 19% more likely to return to homelessness than white residents (Edwards et al., 2021); that is, Black PEH who are approved for PSH may be more likely to never actually exit homelessness and it is not clear that Homeless Management Information Systems (HMIS), which was used in the previous research, captures this information. The finding that among those who received PSH, Black PEH were more likely to live in PB-PSH vs. SS-PSH also deserves further attention as it may suggest a different pattern than previous studies that found Black PEH had greater odds of receiving PSH with lower levels of support services (Dickson-Gomez et al., 2021). Our findings may reflect racial discrimination in the housing market in which private landlords are less likely to accept Black tenants in SS-PSH. While such discrimination is illegal, research has shown it persists and can be difficult to document (Christensen et al., 2021). The fact that PEH needing pet accommodations were also more likely to be assigned to PB-PSH versus SS-PSH suggests that landlord preferences may play an important factor. Of course, both findings discussed above may also reflect our specific sample that while largely representative of all PSH placements during the pandemic was nevertheless a convenience sample.

The findings from this study also suggest that while there is no formal guidance on whether PB- and SS-PSH should be considered a different type of resource or intervention model, homeless service systems may consider PB-PSH more appropriate for PEH with higher needs (Dickson-Gomez et al., 2020). Indeed, we found that individuals with mental health conditions or alcohol use disorders were more likely to be placed in PB-PSH. This is consistent with previous literature on PSH services in the Los Angeles homeless services system (Henwood et al., 2018), although research has demonstrated that intensive service models can also be delivered in the context of SS-PSH (Aubry et al., 2016).

Our results also suggest that there is an interplay between clients’ needs and beliefs in the housing placement process, which involve both predisposing and need factors from the Gelberg-Anderson model. For example, PEH who reported having children younger than 18 years old were more likely to be assigned a PSH unit, which may reflect a willingness of families to accept any housing option that is offered or the homeless service system’s effort to more quickly accommodate families into permanent housing during the pandemic. Participants who were foreign-born were also less likely to be assigned a housing unit, which may reflect a lack of culturally and language responsive services, fears about documentation status and public charge policies, and PEH being unable to access certain housing and public benefits based on documentation status, which is a growing policy issue given a significant rise in the Latino homeless population since the beginning of the pandemic (Los Angeles Homeless Service Authority, 2022). PEH who reported physical health conditions were more likely to be assigned a housing unit, which may reflect a greater recognition of the need for housing among these individuals and the homeless service system, which prioritizes health vulnerability (Cronley, 2022). At the same time, PEH who were homeless the longest (more than 20 years) were less likely to be assigned a housing unit, which may suggest some PEH are highly vulnerable and require additional support in the housing process or that these individuals are more discerning or rigid in exercising their housing preferences. The latter conclusion may be bolstered by the fact that individuals with greater knowledge, skills, and confidence in managing personal health were less likely to have been assigned to any PSH. Interventions that offer more intensive services such as critical time intervention (Ponka et al., 2020) may be better able to help individuals navigate the housing process, as would a more flexible menu of housing options.

Just as housing needs and preference appeared to influence PSH assignment, these factors also appear to influence who ends up in PB- vs. SS-PSH. Those who expressed a need to live in a particular neighborhood were less likely to be assigned to PB-PSH, which underscores that SS-PSH may provide greater choice in neighborhood location (Wong et al., 2007). Housing choice and preferences seemed to play a role in the overall housing process, given those with higher greater knowledge, skills, and confidence in managing personal health were both less likely to be placed into any housing at all and more likely to be placed into PB-PSH. This may suggest that people with stronger preferences have greater difficulty finding a location where they can use their SS-PSH vouchers or more willingness to wait for a PB-PSH unit. Either way, it underscores the importance of exercising choice in services and housing situations that have been shown to be predictive of better outcomes (Greenwood et al., 2005). It is not clear the extent to which current housing systems can accommodate PEH choice or preferences for PSH. But patient-centered care and consumer-driven services have been a cornerstone of health care reform (Berwick, 2009; Epstein et al., 2010) and should also guide homeless services and placement.

### Strengths and Limitations

A notable strength of this study is that it is one of only a few to consider PEH characteristics associated with differences between PB- and SS-PSH placement (Dickson-Gomez et al., 2020, 2021) and did so during a global pandemic. As noted, our sample is largely representative of all people placed in PSH during the pandemic but is nevertheless a convenience sample. As we learned through this study, the Los Angeles County coordinated entry system does not provide any mechanism for contacting clients at the time of enrollment into PSH or indeed even any systemwide view of how many PSH placements would be occurring through particular housing providers over a given time horizon. Consequently, recruitment for this study had to take place through a subset of individual organizations within the Los Angeles County homeless service system. Even within these providers, enrollment was likely significantly less than 100% given substantial turnover of housing program case workers (who were responsible for referring PEH to the study), high client-to-case worker ratios, and pandemic-related barriers to successfully contacting PEH that may have affected who was notified about the study.

Other notable limitations include our relatively small sample sizes, which may limit our ability to detect statistical significance for some differences. Nevertheless, the findings that PEH who received PSH were more likely to lower health activation scores and that PEH who received PB- versus SS-PSH were more likely to Black remained significant even after Bonferroni correction, suggests that these findings may be most salient. Our sample also included individuals who received rapid rehousing if they also received support services, which may have impacted the findings. Finally, a major limitation is that our study lacked information about the decision-making processes or mechanisms that affected housing placement and housing type, which appear to be fruitful areas of future research based on our findings.

## Conclusions

Overall, our findings illustrate the need to clarify whether PB- and SS-PSH should be considered the same or a distinct housing resource, and suggest the need to track whether PEH approved for PSH actually receive PSH in homeless management information systems. We found that PEH with higher needs were more likely to be placed in PB- vs. SS-PSH and more likely to be placed into housing at all. We also found that race may play an important factor in determining whether someone actually receives housing and the type of PSH that they eventually receive; future research in needed to understand this finding and to develop interventions to mitigate it. Finally, our results demonstrate the challenges of conducting comparative effectiveness research on service models that can best support PEH including PSH models (Dickson-Gomez et al., 2020).

## Electronic Supplementary Material

Below is the link to the electronic supplementary material.

Supplementary Material 1

Supplementary Material 2

Supplementary Material 3

## References

[CR1] Aubry, T., Goering, P., Veldhuizen, S., et al. (2016). A multiple-city RCT of Housing First with assertive community treatment for homeless canadians with serious mental illness. *Psychiatric Services*, *67*(3), 275–281. 10.1176/appi.ps.201400587. 26620289 10.1176/appi.ps.201400587

[CR2] Berwick, D. M. (2009). What ‘patient-centered’ should mean: Confessions of an extremist. *Health Affairs*, *28*(Suppl. 1), w555–w565. 10.1377/hlthaff.28.4.w555. 19454528 10.1377/hlthaff.28.4.w555

[CR3] Bovin, M. J., Kimerling, R., Weathers, F. W., et al. (2021). Diagnostic accuracy and acceptability of the primary care posttraumatic stress disorder screen for the Diagnostic and Statistical Manual of Mental Disorders (fifth edition) among US veterans. *JAMA Network Open*, *4*(2), e2036733. 10.1001/jamanetworkopen.2020.36733. 33538826 10.1001/jamanetworkopen.2020.36733 PMC7862990

[CR4] Brown, M., Cummings, C., Lyons, J., Carrión, A., & Watson, D. P. (2018). Reliability and validity of the vulnerability index-service prioritization decision assistance Tool (VI-SPDAT) in real-world implementation. *Journal of Social Distress and the Homeless*, *27*(2), 110–117. 10.1080/10530789.2018.1482991. 10.1080/10530789.2018.1482991

[CR8] Christensen, P., Sarmiento-Barbieri, I., & Timmins, C. (2021). *Racial discrimination and housing outcomes in the United States rental market (no. w29516)*. National Bureau of Economic Research.

[CR9] Cronley, C. (2022). Invisible intersectionality in measuring vulnerability among individuals experiencing homelessness – critically appraising the VI-SPDAT. *Journal of Social Distress and Homelessness*, *31*(1), 23–33. 10.1080/10530789.2020.1852502. 10.1080/10530789.2020.1852502

[CR13] Dickson-Gomez, J., Quinn, K., McAuliffe, T., Bendixen, A., & Ohlrich, J. (2020). Placement of chronically homeless into different types of permanent supportive housing before and after a coordinated entry system: The influence of severe mental illness, substance use disorder, and dual diagnosis on housing configuration and intensity of services. *Journal of Community Psychology*, *48*(7), 2410–2427. 10.1002/jcop.22428. 32789923 10.1002/jcop.22428

[CR12] Dickson-Gomez, J., McAuliffe, T., Quinn, K., et al. (2021). The comparative effectiveness of different models of permanent supportive housing on problematic substance use, depression and anxiety symptoms over time. *American Journal of Orthopsychiatry*, *91*(4), 514–523. 10.1037/ort0000550. 33939448 10.1037/ort0000550 PMC8370390

[CR14] Edwards, E., Milburn, N., Obermark, D., & Rountree, J. (2021). *Inequity in the permanent supportive housing system in Los Angeles: Scale, scope and reasons for black residents’ returns to homelessness*. *California Policy Lab*. https://www.capolicylab.org/inequity-in-the-psh-system-in-los-angeles/.

[CR15] Epstein, R. M., Fiscella, K., Lesser, C. S., & Stange, K. C. (2010). Why the nation needs a policy push on patient-centered health care. *Health Affairs*, *29*(8), 1489–1495. 10.1377/hlthaff.2009.0888. 20679652 10.1377/hlthaff.2009.0888

[CR16] Gelberg, L., Andersen, R. M., & Leake, B. D. (2000). The behavioral model for vulnerable populations: Application to medical care use and outcomes for homeless people. *Health Services Research*, *34*(6), 1273–1302. 10654830 PMC1089079

[CR18] Greenwood, R. M., Schaefer-McDaniel, N. J., Winkel, G., & Tsemberis, S. J. (2005). Decreasing psychiatric symptoms by increasing choice in services for adults with histories of homelessness. *American Journal of Community Psychology*, *36*(3–4), 223–238. 10.1007/s10464-005-8617-z. 16389497 10.1007/s10464-005-8617-z

[CR20] Henwood, B. F., Harris, T., Woo, D., Winetrobe, H., Rhoades, H., & Wenzel, S. L. (2018). Availability of comprehensive services in permanent supportive housing in Los Angeles. *Health and Social Care in the Community*, *26*(2), 207–213. 10.1111/hsc.12510. 28984074 10.1111/hsc.12510 PMC6277053

[CR21] Henwood, B. F., Kuhn, R., Padwa, H., et al. (2023). Investigating the comparative effectiveness of place-based and scatter-site permanent supportive housing for people experiencing homelessness during the COVID-19 pandemic: Protocols for a mixed methods, prospective longitudinal study. *JMIR Research Protocols*, *12*, e46782. 10.2196/46782. 37115590 10.2196/46782 PMC10150866

[CR24] Kuhn, R., Henwood, B., Lawton, A., & Chien, J. (2022). *Under threat: Surveying unhoused Angelenos in the era of camping enforcement*. Price Center for Social Innovation. https://socialinnovation.usc.edu/homeless_research/under-threat-surveying-unhoused-angelenos-in-the-era-of-camping-enforcement/.

[CR27] Los Angeles Homeless Services Authority (2022). *Hispanic/Latino HC22 data summary*. https://www.lahsa.org/documents?id=6588-hispanic-latino-hc22-data-summary.

[CR29] National Academies of Sciences, Engineering, and Medicine. (2018). *Permanent supportive housing: Evaluating the evidence for improving health outcomes among people experiencing chronic homelessness*. National Academies. 10.17226/25133. 30148580

[CR31] Ponka, D., Agbata, E., Kendall, C., et al. (2020). The effectiveness of case management interventions for the homeless, vulnerably housed and persons with lived experience: A systematic review. *PLOS One*, *15*(4), e0230896. 10.1371/journal.pone.0230896. 32271769 10.1371/journal.pone.0230896 PMC7313544

[CR33] Prins, A., Bovin, M. J., Smolenski, D. J., et al. (2016). The primary care PTSD screen for DSM-5 (PC-PTSD-5): Development and evaluation within a veteran primary care sample. *Journal of General Internal Medicine*, *31*(10), 1206–1211. 10.1007/s11606-016-3703-5. 27170304 10.1007/s11606-016-3703-5 PMC5023594

[CR35] Richter, D., & Hoffmann, H. (2017). Preference for independent housing of persons with mental disorders: Systematic review and meta-analysis. *Administration and Policy in Mental Health and Mental Health Services Research*, *44*(6), 817–823. 10.1007/s10488-017-0791-4. 28160182 10.1007/s10488-017-0791-4

[CR38] Somers, J. M., Moniruzzaman, A., Patterson, M., et al. (2017). A randomized trial examining Housing First in congregate and scattered site formats. *PLOS One*, *12*(1), e0168745. 10.1371/journal.pone.0168745. 28076358 10.1371/journal.pone.0168745 PMC5226665

[CR39] Ward, J. M., Garvey, R., & Hunter, S. B. (2022). *Recent trends among the unsheltered in three Los Angeles neighborhoods: An interim report on the Los Angeles Longitudinal Enumeration and demographic survey (LA LEADS) project*. RAND Corporation. https://www.rand.org/pubs/research_reports/RRA1890-1.html. 10.7249/rhq-12-01-11 PMC11630103 39736912

[CR40] Wong, Y. L. I., Filoromo, M., & Tennille, J. (2007). From principles to practice: A study of implementation of supported housing for psychiatric consumers. *Administration and Policy in Mental Health and Mental Health Services Research*, *34*(1), 13–28. 10.1007/s10488-006-0058-y. 16755391 10.1007/s10488-006-0058-y

