# Supplementary material for: Placement into Scattered-Site or Place-Based Permanent Supportive Housing in Los Angeles County, CA, During the COVID-19 Pandemic
Source: Adm Policy Ment Health. 2024 Mar 14;51(5):805–17. doi: 10.1007/s10488-024-01359-1 (PMC11379792; doi:10.1007/s10488-024-01359-1)
Supplement: Supplementary file 1 — Supplementary Material 1 [file 10488_2024_1359_MOESM1_ESM.docx]

**Table A. Survey items and response options as presented to participants**

| Dimension | Item or measure | Response options | Level of measurement |
| --- | --- | --- | --- |
| *Predisposing factors* |  |  |  |
| Demographics |  |  |  |
| Age | In what year were you born? | Open response | Continuous |
| Gender identity | What is your gender identity? | 1. Man 2. Woman 3. Another gender identity | Categorical:   - Man - Woman or other |
| Sexual orientation | Do you consider yourself to be: | 1. Heterosexual or straight 2. Gay or Lesbian 3. Bisexual 4. Another sexual orientation | Categorical:   - Heterosexual or straight - LGB+ (gay, lesbian, bisexual, or another sexual orientation) |
| Relationship status | What best describes your current relationship status? | 1. Single, never married 2. Married or domestic partnership 3. Married but separated 4. Divorced 5. Widowed | Categorical:   - Single - Married or domestic partnership - Separated, divorced, or widowed |
| Veteran status | Have you served in the US military or armed forces? | 1. Yes 2. No | Binary:   - No - Yes |
| Criminal justice involvement | Have you ever spent time in jail, prison, or juvenile detention center? | 1. Yes 2. No | Categorical:   - No involvement - Prior involvement (but not currently on probation or parole) - Currently on probation or parole |
|  | Are you currently on probation or parole? | 1. Yes, on probation 2. Yes, on parole 3. Not on probation or parole |  |
| Social structure |  |  |  |
| Race and ethnicity | What best describes your race/ethnicity? (Select one) | 1. Black/African American 2. White (non-Hispanic/Latino) 3. Hispanic/Latino 4. Asian American 5. Native Hawaiian or Pacific Islander 6. Native American or Alaska Native 7. Multiracial/multiethnic 8. Other | Categorical:   - Black orAfrican American - White (non-Hispanic or Latino) - Hispanic or Latino - Multiracial or other (Asian American, Native Hawaiian, Pacific Islander, Native American, or Alaska Native) |
| Country of birth | In what country were you born? | 1. US-born 2. Foreign-born | Binary:   - U.S.-born - Foreign-born |
| Educational attainment | What is the highest level of education you received? | 1. 8^th^ grade or less 2. Some high school 3. High school or GED 4. Some college, no degree 5. Associates degree or vocational certificate 6. Bachelor’s degree or higher | Categorical:   - Less than high school - Some college, no degree - Associate or bachelor’s degree or higher |
| Employment status | What is your employment status? | 1. Employed, full-time (+35 hours/week) 2. Employed, part-time; Unemployed, looking for work 3. Unemployed, not looking for work 4. Retired, receiving retirement benefits 5. Retired, not receiving benefits | Categorical:   - Employed full- or part-time - Unemployed - Retired |
| Coresiding children < 18 years old | Do you have any children under the age of 18? | 1. Yes 2. No | Binary:   - No - Yes |
| Years homeless | How many months have you experienced homelessness in your life? | (Open response) | Categorical:   - Less than 5 years - 5–9 years - 10–19 years - 20 or more |
| History of unsheltered homelessness | Think about your whole life. Which of these places have you ever stayed in?  (Select all that apply.) | 1. Abandoned building 2. Bus, subway, or train 3. Car, truck, van, or RV 4. Garage or shed not meant for living in 5. Indoor public place (all-night theater, restaurant, train/bus station) 6. Outside on the street, park, or beach 7. Temporary or emergency shelter 8. None of these | Binary:   - No - Yes |
| Unsheltered homelessness during month before COVID-19 pandemic onset | In the month leading up to the COVID-19 pandemic in March 2020, where did you usually sleep? [select all that apply] | 1. Shelter, emergency/temporary housing, or hotel provided by an agency 2. Outside 3. Tent or makeshift shelter 4. In a bus station, train station, or airport 5. Abandoned building 6. Vehicle (car, van, truck, RV) 7. My own apartment or home 8. Hotel that I paid for 9. Institution, hospital, or correctional facility | Binary:   - No - Yes |
| Health beliefs |  |  |  |
| Health activation | Insignia Health Patient Activation Measure   1. Taking an active role in my own healthcare is the most important thing that affects my health. 2. I know how to prevent problems with my health. 3. I can figure out solutions when new problems arise with my health. 4. I can maintain lifestyle changes like eating right and exercising, even when things are stressful | 1. Strongly disagree 2. Disagree 3. Agree 4. Strongly agree | Continuous: mean of four items; possible range of 0­–4, with higher scores indicating greater health activation |
| Housing preferences | How would you prefer to live? | 1. In an apartment alone (or just with your partner/spouse/family member) 2. With a roommate (other than a partner/spouse/family member) 3. No preference | Categorical:   - In an apartment alone (or just with partner, spouse, or family member) - With a roommate (other than a partner, spouse, or family member) - No preference |
|  | Would you prefer to live in a building where: | 1. Most of the other residents in the building have experienced homelessness 2. Most of the other residents in the building have not experienced homelessness 3. No preference | Categorical:   - Most other residents in the building have experienced homelessness - Most other residents in the building have not experienced homelessness - No preference |
|  | Would you prefer to live in a building that | 1. Requires sober living and abstinence 2. Permits the use of alcohol and drugs 3. Does not have any rules about alcohol and drugs 4. I don’t have a preference | Binary:   - No (i.e., permits use of alcohol; does not have any rules; or no preference) - Yes (i.e., requires sober living and abstinence) |
|  | Would you prefer to live in a setting where everyone is the same sex/gender as you? | 1. Yes 2. No 3. I don’t have a preference | Binary:   - No or no preference - Yes |
| *Enabling factors* |  |  |  |
| Government benefits | Which of the following benefits do you currently receive? | 1. General Relief (GR), Temporary Cash Assistance (TCA), Family Independence Temporary Assistance (FITAP), or Family Investment Program (FIP) 2. Social Security Disability Income (SSDI) or Social Security Income (SSI) 3. Retirement Outcome 4. Supplemental Nutritional Assistance (SNAP)/CalFresh/WIC 5. Temporary Assistance for Needy Families (TANF) 6. Veterans’ Benefits 7. CalWORKS 8. Unemployment Benefits 9. COVID-19 Paycheck Protection/Cash Relief 10. Other 11. None of the above | Binary:   - No - Yes |
| Monthly income | Approximately how much is your total monthly income? | Open response | Categorical:   - Less than $500 - $500^–^$999 - $1,000–$1,999 - $2,000 or more |
| Health insurance | Do you have health insurance that helps pay for some or all of your healthcare costs? | 1. Yes 2. No | Binary:   - No - Yes |
| *Need factors* |  |  |  |
| Past chronic physical disease diagnosis | Have you ever been told by a doctor or other health professional that you have any of the following? (Select all that apply.) | 1. Asthma 2. COPD or other chronic respiratory disease 3. Diabetes 4. Hypertension 5. Chronic kidney disease 6. Liver disease (e.g. hepatitis; cirrhosis) 7. Heart condition 8. Neurodegenerative disease (e.g. Parkinson’s, MS) 9. Blood disorder (e.g. sickle cell disease) 10. Cancer 11. HIV/AIDS 12. Other health condition 13. None of the above | Binary:   - No - Yes |
| Past mental health condition diagnosis | Have you ever been told by a doctor or other health professional that you have any of the following? (Select all that apply.) | 1. Major depression 2. Anxiety 3. Bipolar disorder 4. Schizophrenia or other psychotic disorders not related to substance use 5. PTSD 6. Other mental health disorders 7. None of the above | Binary:   - No - Yes |
| Past substance use disorder diagnosis | Have you ever been told by a doctor or other health professional that you have any of the following? | 1. Alcohol use disorder 2. Cannabis/marijuana use disorder 3. Stimulant (methamphetamine/cocaine) use disorder 4. Opioid (heroin, fentanyl, prescription opioid) use disorder 5. Other substance use disorders (not including tobacco) 6. None of the above | Binary:   - No - Yes |
| Trauma history | PC-PTSD-5 (Prins)  Sometimes things happen to people that are unusually or especially frightening, horrible, or traumatic. For example:   - A serious accident or fire - A physical or sexual assault or abuse - An earthquake or flood - A war - Seeing someone be killed or seriously injured - Having a loved one die through homicide or suicide  1. Have you ever experienced this kind of event?   If responded “yes” to item above:   1. In the past month, have you had nightmares about these events or thought about these events when you did not want to? 2. In the past month, have you tried hard not to think about the event(s) or gone out of your way to avoid situations that reminded you of the event(s)? 3. In the past month have you been constantly on guard, watchful, or easily startled? 4. In the past month, have you felt numb or detached from people, activities, or your surroundings? 5. In the past month, have you felt guilty or unable to stop blaming yourself or others for the event(s) or any problems the event(s) may have caused? | 1. Yes 2. No | Categorical:   - No trauma experience or PTSD symptoms - Experienced trauma but no PTSD symptoms - Experienced trauma and PTSD symptoms   Presence of PTSD calculated by summing items b–f; scores of 3 or higher denote “probable PTSD”) |
| Housing needs | Which of the following specific needs or requirements for housing do you have? (Select all that apply). | 1. A lot of space to store possessions 2. Allowed to stay with pets 3. Allowed to stay with partner, spouse, or family member 4. Handicap accessible 5. Having my own private bathroom 6. Live with other Veterans 7. Needs to be in a particular neighborhood 8. Other 9. None of the above | Binary:   - No - Yes |
