# Supplementary material for: Placement into Scattered-Site or Place-Based Permanent Supportive Housing in Los Angeles County, CA, During the COVID-19 Pandemic
Source: Adm Policy Ment Health. 2024 Mar 14;51(5):805–17. doi: 10.1007/s10488-024-01359-1 (PMC11379792; doi:10.1007/s10488-024-01359-1)
Supplement: Supplementary file 2 — Supplementary Material 2 [file 10488_2024_1359_MOESM2_ESM.docx]

**Table B. Comparison of the composition of PCHOOSE sample to all PSH placements recorded in Los Angeles County administrative records (April 2021–September 2022)**

| Characteristic | PB-PSH | | | | SS-PSH | | |
| --- | --- | --- | --- | --- | --- | --- | --- |
|  | L.A. County | PCHOOSE | | | L.A. County | PCHOOSE | |
|  | (*n* = 4,414) | (*n* = 272) | | | (*n* = 2,817) | (*n* = 185) | |
|  | *M* | *M* | 95% CI | *M* | | *M* | 95% CI |
| Gender |  |  |  |  | |  |  |
| Female | 37% | 37% | 31%, 43% | 40% | | 49% | 41%, 56% |
| Male | 62% | 61% | 55%, 66% | 59% | | 51% | 43%, 58% |
| Other or unknown | 1% | 3% | 1%, 5% | 0% | | 1% | 0%, 3% |
| Race and ethnicity |  |  |  |  | |  |  |
| Non-Hispanic Black | 40% | 42% | 36%, 48% | 43% | | 30% | 24%, 38% |
| Non-Hispanic White | 21% | 20% | 15%, 25% | 18% | | 34% | 27%, 41% |
| Hispanic or Latino | 30% | 24% | 19%, 30% | 29% | | 25% | 19%, 32% |
| Other or unknown | 10% | 14% | 10%, 19% | 9% | | 11% | 7%, 16% |
| Age (years) |  |  |  |  | |  |  |
| 30 or younger | 17% | 12% | 9%, 17% | 16% | | 17% | 12%, 24% |
| 31–40 | 16% | 18% | 14%, 23% | 18% | | 26% | 20%, 34% |
| 41–50 | 18% | 20% | 15%, 25% | 17% | | 14% | 10%, 20% |
| 51–60 | 25% | 25% | 20%, 31% | 27% | | 24% | 18%, 31% |
| 61–70 | 18% | 21% | 17%, 27% | 18% | | 16% | 11%, 23% |
| 71 or older | 5% | 4% | 2%, 7% | 4% | | 2% | 1%, 6% |

*Note.* PSH = permanent supportive housing; L.A. = Los Angeles; PB-PSH = Place-based permanent supportive housing; SS-PSH = scattered-site permanent supportive housing; CI = confidence interval.
