# Supplementary material for: Placement into Scattered-Site or Place-Based Permanent Supportive Housing in Los Angeles County, CA, During the COVID-19 Pandemic
Source: Adm Policy Ment Health. 2024 Mar 14;51(5):805–17. doi: 10.1007/s10488-024-01359-1 (PMC11379792; doi:10.1007/s10488-024-01359-1)
Supplement: Supplementary file 3 — Supplementary Material 3 [file 10488_2024_1359_MOESM3_ESM.docx]

**Table C. Comparison of predisposing, enabling, and need factors among PCHOOSE sample assigned to scattered-site versus rapid rehousing PSH**

| Characteristic | PSH assignment^a^ | |  |
| --- | --- | --- | --- |
|  | SS-PSH | RR-PSH |  |
|  | (*n* = 71) | (*n* = 114) |  |
|  | *n* (%)^b^ | *n* (%)^b^ | *p*^c^ |
| *Predisposing factors* |  |  |  |
| Demographic characteristics |  |  |  |
| Age (years), *M* (*SD*) | 48.25 (15.48) | 43.87 (13.90) | .05 |
| Gender identity |  |  | .07 |
| Man | 30 (42.25) | 64 (56.14) |  |
| Woman or other | 41 (57.75) | 50 (43.86) |  |
| Sexual orientation |  |  | 0.81 |
| Heterosexual or straight | 61 (88.41) | 94 (86.24) |  |
| LGB+ | 7 (10.14) | 11 (10.09) |  |
| Prefer not to answer | 1 (1.45) | 4 (3.67) |  |
| Relationship status |  |  | 0.24 |
| Single | 44 (62.86) | 68 (60.18) |  |
| Married or domestic partnership | 8 (11.43) | 23 (20.35) |  |
| Separated, divorced, or widowed | 18 (25.71) | 22 (19.47) |  |
| Veteran of the military (yes) | 3 (4.23) | 2 (1.75) |  |
| Criminal justice involvement |  |  | 0.74 |
| None | 29 (41.43) | 53 (47.32) |  |
| Prior involvement | 35 (50.00) | 50 (44.64) |  |
| On parole or probation | 6 (8.57) | 9 (8.04) |  |
| Social structure |  |  |  |
| Race and ethnicity |  |  | 0.24 |
| Black | 22 (30.99) | 34 (29.82) |  |
| White | 29 (40.85) | 33 (28.95) |  |
| Hispanic or Latino | 13 (18.31) | 34 (29.82) |  |
| Other or multiracial | 7 (9.86) | 13 (11.40) |  |
| Foreign-born (yes) | 6 (8.45) | 13 (11.40) | 0.52 |
| Educational attainment |  |  | 0.11 |
| Less than high school | 10 (14.08) | 29 (25.66) |  |
| High school or GED | 21 (29.58) | 39 (34.51) |  |
| Some college | 25 (35.21) | 31 (27.43) |  |
| Associate or bachelor’s degree or higher | 15 (21.13) | 14 (12.39) |  |
| Employment status |  |  | 0.02 |
| Employed full- or part-time | 13 (18.57) | 11 (10.00) |  |
| Unemployed | 45 (64.29) | 91 (82.73) |  |
| Retired | 12 (17.14) | 8 (7.27) |  |
| Co-residing children < 18 years old (yes) | 5 (7.04) | 21 (18.58) | 0.03 |
| Years homeless |  |  | 0.25 |
| Less than 5 | 31 (43.66) | 59 (51.75) |  |
| 5–9 | 18 (25.35) | 34 (29.82) |  |
| 10–19 | 15 (21.13) | 13 (11.40) |  |
| 20 or more | 7 (9.86) | 8 (7.02) |  |
| History of unsheltered homelessness | 68 (97.14) | 97 (87.39) | 0.02 |
| Unsheltered homelessness during month before COVID-19 pandemic onset | 40 (56.34) | 64 (57.14) | 0.91 |
| Health beliefs |  |  |  |
| Health activation score,^d^ *M* (SD) | 2.02 (0.36) | 1.88 (0.44) | 0.05 |
| Living alone or with roommates |  |  | 0.22 |
| Alone or with spouse or family member | 60 (89.55) | 99 (94.29) |  |
| With roommate | 7 (10.45) | 5 (4.76) |  |
| No preference | 0 (0.00) | 1 (0.95) |  |
| Living with homeless residents |  |  | 0.60 |
| Most residents with homeless experience | 7 (10.00) | 17 (15.18) |  |
| Most residents with no homeless experience | 20 (28.57) | 29 (25.89) |  |
| No preference | 43 (61.43) | 66 (58.93) |  |
| Require sober living housing (yes) | 11 (20.00) | 12 (16.00) | 0.55 |
| Requires same gender housing (yes) | 2 (3.64) | 5 (6.67) | 0.70 |
| *Enabling factors* |  |  |  |
| Receive any type of benefits (yes) | 64 (90.14) | 96 (84.21) | 0.25 |
| Monthly income |  |  | 0.09 |
| Less than $500 | 26 (36.62) | 54 (47.37) |  |
| $500–$999 | 20 (28.17) | 28 (24.56) |  |
| $1,000–$1,999 | 17 (23.94) | 29 (25.44) |  |
| $2,000 or more | 8 (11.27) | 3 (2.63) |  |
| Have health insurance (yes) | 69 (98.57) | 99 (86.84) | <0.01 |
| *Need factors* |  |  |  |
| Any physical health condition (yes) | 48 (67.61) | 75 (67.57) | >0.99 |
| Any mental health condition (yes) | 44 (61.97) | 63 (57.27) | 0.53 |
| Any substance use disorder (yes) | 13 (18.31) | 23 (20.18) | 0.76 |
| Trauma history and PTSD symptoms |  |  | 0.40 |
| None | 18 (25.35) | 34 (29.82) |  |
| Trauma but no PTSD symptoms | 24 (33.80) | 30 (26.32) |  |
| Trauma and PTSD symptoms | 18 (25.35) | 38 (33.33) |  |
| Prefer not to answer | 11 (15.49) | 12 (10.53) |  |
| Housing needs (yes) |  |  |  |
| Spouse, partner, or family member allowed | 24 (33.80) | 56 (49.12) | 0.04 |
| Pets allowed | 23 (32.39) | 36 (31.58) | 0.91 |
| Lot of space for possessions | 24 (33.80) | 30 (26.32) | 0.28 |
| Private bathroom | 35 (63.64) | 41 (54.67) | 0.31 |
| Handicap accessible | 16 (22.54) | 25 (21.93) | 0.92 |
| Particular neighborhood | 19 (26.76) | 38 (33.33) | 0.35 |
| Other | 11 (15.49) | 12 (10.53) | 0.32 |
| None of the above | 7 (9.86) | 24 (21.05) | 0.05 |
| Prefer not to answer | 1 (1.41) | 5 (4.39) | 0.41 |

*Note.* LGB+ = lesbian, gay, bisexual, or other sexual orientation; PSH = permanent supportive housing; RR-PSH = rapid rehousing permanent supporting housing; SS-PSH = scattered-site permanent supportive housing. Percentages may not add to 100 due to rounding.

^a^ As specified at the time of enrollment.

^b^ Unless otherwise noted.

^c^ Kruskal-Wallis rank sum test, Fisher’s exact test for count data with simulated *p*-value (based on 10,000 replicates), or Pearson’s chi-square test.

^d^ Possible health activation scores range from 0–3, with higher values indicating greater patient activation.
